# Calcium-binding protein 39 overexpression promotes macrophages from ‘M1’ into ‘M2’ phenotype and improves chondrocyte damage in osteoarthritis by activating the AMP-activated protein kinase/sirtuin 1 axis

**DOI:** 10.1080/21655979.2022.2061289

**Published:** 2022-04-12

**Authors:** Qiuliang Liu, Kai Pian, Zhen Tian, Haitao Duan, Qi Wang, Hui Zhang, Longyan Shi, Dongjian Song

**Affiliations:** Department of Pediatric Surgery, The First Affiliated Hospital of Zhengzhou University, Zhengzhou Henan, China

**Keywords:** CAB39, macrophages, osteoarthritis, AMPK, sirt-1

## Abstract

Osteoarthritis (OA) is a degenerative joint disease that affects cartilage and its peripheral tissues. Up-regulation of Calcium-binding protein 39 (CAB39) has a significant protective effect on osteoblasts, but the role and related molecular mechanisms of CAB39 in OA have not yet been reported. CAB39 overexpression and knockdown models were set up in chondrocytes (ATDC5) and macrophages (RAW264.7). The OA cell model was induced in ATDC5 cells with IL-1β (10 ng/mL). Cell viability was tested by the cell counting kit-8 assay, apoptosis was checked by flow cytometry. Western blot was applied for checking the expression of MMP3, MMP13, Aggrecan, the AMPK/Sirt-1 pathway, apoptosis-related proteins (Bax, Bcl-2, and Caspase-3), and macrophage phenotypic markers (CD86, iNOS, CD206, and Arg1). An OA model was constructed in mice, and CAB39 overexpression plasmids were administered to the knee cavity of the OA model mice. As a result, CAB39 was down-regulated in IL-1β-treated chondrocytes and OA mice. Overexpressing CAB39 enhanced ATDC5 cell viability and choked IL-1β-mediated apoptosis. Overexpression of CAB39 boosted the polarization of macrophages from M1-phenotype into M2 phenotype. In addition, overexpressing CAB39 facilitated the AMPK/Sirt-1 pathway activation, and AMPK inhibitors reversed the protective effect of CAB39 overexpression on chondrocytes. Moreover, CAB39 exhibited anti-inflammatory effects in OA mice by activating the AMPK/Sirt-1 pathway. Collectively, overexpressing CAB39 heightened macrophages’ M2 polarization and declined chondrocyte injury in OA by activating the AMPK/Sirt-1 pathway.**Abbreviations** AMPK: AMP-activated protein kinaseArg1: arginase 1Bax: Bcl-2-associated X proteinBcl-2: B-cell lymphoma-2CAB39: Calcium-binding protein 39CM: Conditioned mediumDMM: destabilization of the medial meniscusECM: extracellular matrixELISA: enzyme-linked immunosorbent assayFCM: Flow cytometryIL-1β: interleukin-1βIL-4: interleukin-4IL-6: interleukin-6IL-10: interleukin-10IFN – γ: Interferon-gammaIHC: ImmunohistochemistryiNOS: Inducible nitric oxide synthaseLKB1: liver kinase B1MMP3: Matrix metalloproteinase3MMP13:Matrix metalloproteinase13NF-κB: NF-kappaBOA: OsteoarthritisqRT-PCR: Quantitative reverse transcription-polymerase chain reactionRT: room temperatureSirt-1: sirtuin 1STRAD: STE20-related adaptor alphaWB: Western blot

## Graphical abstract

**Figure:**
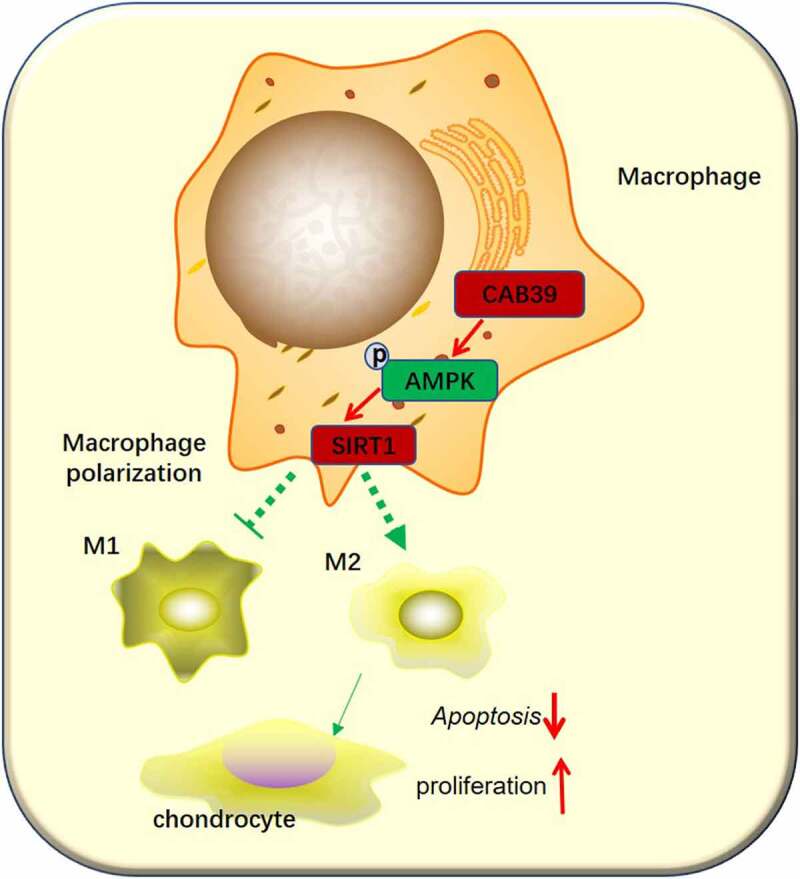

## 1. Introduction

Osteoarthritis (OA) is a chronic joint disorder characterized by degeneration and pain of articular cartilage and is also the main cause of motion-related disability [1,2]. The risk factors of OA mainly include aging, obesity, metabolic diseases, genetic susceptibility, and joint damage [3,4]. The typical clinical manifestations of OA are pain, joint swelling, and joint stiffness [5]. Chondrocyte dysfunction is the main mechanism leading to OA. It has been reported that pro-inflammatory cytokines and oxidative stress induce abnormal proliferation, hypertrophy, and aging of chondrocytes, which contribute to OA evolvement [6].

Macrophages are immune cells that reside within the synovial membrane and can polarize into pro-inflammatory (M1) and anti-inflammatory (M2) phenotypes. Basically, M1-polarized macrophages contribute to OA progression, while M2-polarized macrophages are conducive to chondrogenesis and delay the evolution of OA [7]. M1-polarized macrophage-associated pro-inflammatory cytokines (IL-6, IL-1β, and TNF-α) induce chondrocyte apoptosis through down-regulation of type II collagen and proteoglycan (Aggrecan) synthesis [8]. M2-polarized macrophages, also known as wound-healing macrophages, induce M0 to M2 polarization during OA and are implicated in the repair of injured articular cartilage [9]. In addition, macrophages are activated in OA and the conversion of macrophages from M1 to M2 phenotype is an indication of effective treatment of OA [10]. Hence, regulating macrophage inflammation contributes to ameliorating chondrocyte injury and treating OA.

Calcium-binding protein 39 (CAB39) is a highly conserved protein that is expressed early in mouse embryogenesis. CAB39 is a scaffolding protein for liver kinase B1 (LKB1), and the latter is one of the upstream kinases of AMPK [11]. CAB39 forms a heterotrimeric complex by binding to LKB1 and STRAD, which is required to stabilize LKB1 activity [12,13]. According to reports, heightening the CAB39 expression to activate the AMPK pathway can prevent LPS-induced inflammation and oxidative stress [14]. Besides, oxidative damage and cytotoxicity of dexamethasone to human osteoblasts are alleviated by overexpressing CAB39 [15]. Nonetheless, CAB39’s function in OA and its regulation on macrophages needs further study.

AMP-activated protein kinase (AMPK) is a threonine/serine kinase that is also considered a sensor of the cells’ energy status. When a decrease in ATP production leads to a relative enhancement in AMP or ADP, AMPK is motivated to maintain the balance of ATP synthesis and consumption [16]. Upstream protein kinases, such as LKB1 and CaMMK, activate AMPK through phosphorylation [17]. Activated AMPK facilitates cell survival under stress [18]. Studies have uncovered that the AMPK activity is declined in OA chondrocytes [11]. Metformin impedes cartilage extracellular matrix degradation and boosts chondrocyte synthesis by activating the AMPK pathway [19]. Sirt-1 is a class III histone deacetylase (HDAC) and a nicotinamide adenine dinucleotide (NAD^+^)-dependent enzyme. Sirt-1 catalyzes the deacetylation of histones and non-histones and is thought to be implicated in anti-inflammatory, antioxidant, and anti-apoptotic effects in OA [20]. Sirt-1 is lowly expressed in OA chondrocytes [21]. Activated AMPK modulates energy metabolism through activation of downstream Sirt-1, abates cellular stress and inflammation, and reduces chondrocyte apoptosis [22,23]. However, the mechanism of CAB39 in regulating the AMPK/Sirt-1 pathway in OA remains elusive.

This research was carried out to figure out the function and potential molecular mechanisms of CAB39 in OA. The outcomes exhibited that CAB39 was lowly expressed in the bone and joint tissues of destabilization of the medial meniscus (DMM) model mice. CAB39 overexpression strengthened the polarization of macrophages’ M1 phenotype to M2 phenotype, attenuated macrophage- and IL-1β-induced chondrocyte damage, and up-regulated the AMPK/Sirt-1 axis. From this, we hypothesized that CAB39 slows OA progression through activation of AMPK/Sirt-1 pathway.

## 2. Methods and materials

### 2.1 Cell culture

As mentioned earlier [24], mouse primary chondrocyte cell line (ATDC5) and macrophage cell line (RAW264.7) were bought from Sigma Aldrich (St. Louis, MO). ATDC5 and RAW264.7 cells were grown in the DMEM/F12 medium (Thermo Fisher Scientific, Shanghai, China) comprising 5% fetal bovine serum (Thermo Fisher Scientific, Shanghai, China). The cells were maintained at 37°C in a humidified incubator with 5% CO_2_, with the medium altered every two days. ATDC5 cells were treated with varying concentrations of IL-1β (5, 10, 20, 100 ng/mL) for 24 hours. RAW264.7 cells were treated with LPS (100 ng/mL) + IFNγ (20 ng/mL) and IL-4 (20 ng/mL) + IL-13 (20 ng/mL) for 24 hours, respectively.

After RAW264.7 macrophages were treated with LPS (100 ng/mL) +IFN-γ (20 ng/mL) for 24 hours to induce M1-polarized macrophages, the supernatant of polarized macrophages was harvested via centrifugation (1000 rpm, 5 minutes) and preserved at −80°C for further experiments. Conditioned medium (CM) from differentiated macrophages was diluted with a serum-free medium at a ratio of 1:1 and added to chondrocytes for further culture. LPS (Escherichia coli 0111:B4) was bought from Sigma-Aldrich (St. Louis, MO), while IL-1β, IL-4, IL-13 and IFN-γ were acquired from PeproTech (Rocky Hill, NJ, USA).

### 2.2 Cell transfection

ATDC5 and RAW264.7 cells in good growth state were digested with 0.25% trypsin and seeded into 6-well plates (1 × 10^5^ cells/well). When the cells reached a fusion rate of 80%–90%, they were trypsinized and harvested. Lipofectamine 2000 (ThermoFisherScience, Waltham, MA, USA) was adopted to transfect vectors, CAB39, Si-NC, and Si-CAB39 into ATDC5 and RAW264.7 cells by observing the manufacturer’s guidelines. The transfected cells were kept in a 37°C incubator for 12 hours to observe their status. The serum-free medium was substituted with the complete medium and further incubated for 48 hours. Next, the cellular RNA was separated to verify the transfection validity [25]. Vectors, CAB39, Si-NC, and Si-CAB39 were devised and integrated by GenePharma (Shanghai, China).

### 2.3 Cell counting kit-8 (CCK-8) assay

CCK8 was employed to examine cell proliferation as described previously [26]. Stably transfected ATDC5 cells were taken, trypsinized, adjusted to a cell density of 2 × 10^3^ cells/mL, inoculated in a 96-well plate and treated with or without IL-1β (10 ng/mL) and BML-275 (5 μM) for 24 hours. ATDC5 cells in another plate were treated with the CM (500 μL) of macrophages for 24 hours. After 24 hours of adherent culture, 10 μL of CCK-8 (Yeasen Biotech Co, Ltd.) solution was added to each well. The cells were incubated for one hour, and the absorbance value at 450 nm was reviewed at 24 hours with a microplate reader (Bio-Rad, Hercules, CA, USA). BML-275 (also known as Dorsomorphin or Compound C), an AMPK inhibitor [27], was bought from MedChemExpress. A solution with a concentration of 5 mM was prepared by dissolving 5 mg of BML-275 in 2.5032 mL of DMSO according to the manufacturer’s protocol. When used, the solution was proportionally diluted to 5 μM.

### 2.4 Caspase3 activity detection

Forty-eight hours after transfection, ATDC5 cells were trypsinized, rinsed with phosphate buffer, centrifuged, collected, inoculated in a 6-well plate (2 × 10^6^ cells/well) and treated with or without IL-1β (10 ng/mL) and BML-275 for 24 hours. ATDC5 cells in another 6-well plate were processed with the CM of macrophages for 24 hours. Followed by washing with PBS (cold) three times, ATDC5 cells were added with lysis buffer on ice for 15 min. The lysate was collected, centrifuged at 16,000 g at 4 C for 15 min. We then determined caspase-3 activity using Caspase 3 Activity Assay Kit (Beyotime, Shanghai, China). The release of p-nitroanilide (pNA) was qualified by determining the absorbance with Multiskan Spectrum (Thermo) at 405 nm [28].

### 2.5 Quantitative reverse transcription-polymerase chain reaction (qRT-PCR)

As per the manufacturer’s solution (Invitrogen, Waltham, MA, USA), the total cellular RNA was separated by utilizing the TRIzol reagent. RNA content and purity were tested by Nanodrop-spectrophotometer. We applied the PrimeScript-RT Kit (Madison, WI, USA) to synthesize complementary DNA (cDNA) from 1 µg of total RNA as per the manufacturer’s protocol. We then employed SYBR®Premix-Ex-Taq™ (Takara, TX, USA) and an ABI7300 system for qRT-PCR. The total volume of the PCR system was 30 µL, and each sample included 300 ng cDNA. The amplification was made with an initial denaturation at 95°C for 10 minutes followed by 45 cycles of 95°C for 10 seconds, 60°C for 30 seconds and 85°C for 20 seconds. The relative expression of the gene was calculated by the 2^−ΔΔCt^ method [29], with GAPDH as the housekeeping gene for CAB39. qRT-PCR was made three times. The specific primer sequences are as follows:

CAB39 forward 5’-CTGCGTCATCTTCCTTCAGC-3’; reverse 5’-GCTGGGTTCATGAAGGCAAA-3’.

GAPDH forward 5’-TGGTTGAGCACAGGGTACTT-3’; reverse 5’-CCAAGGAGTAAGACCCCTGG-3’.

### 2.6 Western blot (WB)

WB was conducted as per previous studies [30]. After RAW264.7 and ATDC5 cells and cartilage tissues were exposed to different factors, total cellular and tissue proteins were isolated with pre-cooled RIPA lysate (Beyotime Biotechnology, Shanghai, China). Following centrifugation, the supernatant was taken, and the protein was quantified with the bicinchoninic acid (BCA) protein quantification kit (Beyotime Biotechnology, Shanghai, China). Proteins (30 μg) in each group were subjected to 12% sodium dodecyl sulfate-polyacrylamide gel electrophoresis (SDS-PAGE) (Thermo Fisher Scientific, CA, USA) for 1.5–2 hours. Proteins on the gel were transferred to polyvinylidene fluoride (PVDF) membranes (Millipore, Bedford, MA, USA). After being sealed at RT using TBST solution containing 3% BSA, the membranes were maintained with the primary antibodies of anti-CAB39 (ab51132, Abcam, 1:5000), anti-MMP3 (ab52915, Abcam, 1:1000), anti-MMP13 (ab51072, Abcam, 1:1000), anti-Aggrecan (PA1-1746, Thermo Fisher, 1:1000), anti-p-AMPKα (PA5-17,831, Thermofisher, 1:1000), anti-Sirt-1 (ab189494, Abcam, 1:1000), anti-Bax (ab32503, Abcam, 1:2000), anti-Bcl-2 (ab182858, Abcam, 1:2000), anti-C-Caspase-3 (ab2302, Abcam, 1:500), anti-CD86 (ab239075, Abcam, 1:1000), anti-iNOS (ab178945, Abcam, 1:1000), anti-CD206 (ab252921, Abcam, 1:1000), anti-Arg1 (ab133543, Abcam, 1:1000) and anti-β-actin (ab8227, Abcam, 1:1000) overnight at 4°C. After that, the membranes were cleaned five times (3 minutes each time) with TBST. Next, they were kept at RT with the fluorescein-labeled goat anti-rabbit secondary antibody (ab6721, Abcam, 1:2000) for one hour and then flushed six times with TBST for 3 minutes each time. Finally, the membranes underwent ECL (Amersham Pharmacia Biotech, Little Chalfont, UK) developing, fixing, and scanning. ImageJ was employed to determine the gray intensity of each band, and the ratio of the gray value of the target protein to the gray value of β-actin was the relative protein expression.

### 2.7 Animal experiments

According to a previous study [31], the OA model was induced by destabilization of the medial meniscus (DMM) surgery using adult male C57BL/6 mice. The mice were housed in a specific pathogen-free environment with a 12-hour light/dark cycle that allowed free access to water and food. After anesthesia with an intraperitoneal injection of sodium phenobarbital (100 mg/kg), the right knee joint of the mice (8-week-old, 25 ± 2 g, n = 20 for each group) was exposed via a medial capsulotomy, and then the medial meniscus was displaced medially by severing the tibial ligament of the medial meniscus. At last, the incision was sutured and the skin closed. In the sham group, only the skin of the right knee was removed. Mice were executed 4 weeks after surgery, and their right knee joints were taken for qRT-PCR and histological examination. For the treatment group, 8-week-old male C57BL/6 mice were randomly divided into the sham, OA, OA+LV-NC, and OA+LV-CAB39 groups (6 mice per group). Lentiviral vectors were administered to the right knee cavity of the mice (the sham group was injected with an equivalent dose of saline) once a week for 4 weeks. The mice were euthanized with an intraperitoneal injection of an overdose of sodium pentobarbital at the fourth week after the first injection. The mice’s blood and knee joints were gathered for ELISA and histological analysis. All animal experiments were endorsed by the Animal Ethics Committee of the First Affiliated Hospital of Zhengzhou University (Approval number: FAHZU2021-045). All experimental procedures were carried out in accordance with the Regulations on the Administration of Laboratory Animals approved by the State Council of the People’s Republic of China. Extensive efforts have been made to minimize the number and suffering of animals used in experiments.

### 2.8 Enzyme-linked immunoadsorbent assay (ELISA)

The collected mice blood was left for 15 minutes, centrifuged at 3000 rpm at 4°C for 20 minutes, and then the serum was separated and conserved at −80°C. The contents of IL-6, IL-1β and IL-10 in the isolated serum were determined with ELISA kits (Abcam, Shanghai, China) following the manufacturer’s instructions [32].

### 2.9 Immunohistochemistry (IHC)

The mice’s right knee tissues were secured in 4% paraformaldehyde for 24 hours, decalcified in 12.5% ethylenediaminetetraacetic acid (EDTA) (pH 7.4) for four weeks, dehydrated with graded concentrations of ethanol and paraffin-embedded. The entire medial compartment of the joint was cut on a sagittal section (5 μM thick), which was dewaxed in xylene. After thermal repair of the antigen based on the type of primary antibodies, the endogenous peroxidase was inactivated with 3% H_2_O_2_. Next, sections were blocked with 10% normal goat serum and maintained with anti-Caspase-3 (ab32351, Abcam, 1:100), anti-CAB39 (ab51132, Abcam, 1:100), anti-AMPK (ab32047, Abcam, 1:100) and anti-Sirt-1 (ab189494, Abcam, 1:500) overnight at 4°C. Afterward, they were flushed with PBS and then incubated with biotin-conjugated goat anti-rabbit IgG (A16177, Thermo Fisher, 1:100) secondary antibody for 1 hour at room temperature. The sections were washed again, dyed with 3,3-diaminobenzidine hydrochloride for 1 minute, flushed with double distilled water, stained with hematoxylin for 1 minute and observed under the microscope. Images were acquired with a FluoView FV1000 confocal microscope (Olympus, Tokyo, Japan). The expression level Caspase3, AMPK, and Sirt1 in cartilage tissue was determining by using Image-Pro Plus (Media Cybernetics, Inc., US). Briefly, the average intensity of optical density (also called as IOD/mm^2^) was here defined as sum of integrated option density divided by area of cartilage tissue in the ROI under a magnification of 40 **× **. These procedures were performed [33].

### 2.10 Statistical analysis

Statistical analysis was carried out by utilizing the GraphPad Prism 8.0 software (GraphPad Software Inc., La Jolla, CA, USA). Results were represented as mean ± SD. A *t-*test was employed to analyze the statistical significance between the groups, with *P* < 0.05 being a statistically significant difference.

## 3. Results

In this study, we sought to study the impact of CAB39 on the macrophage polarization and the effect of activated macrophage on chondrocyte damage in osteoarthritis. The polarization of the macrophages was verified after CAB39 regulation. In-vitro OA model was constructed on chondrocytes by the conditioned medium of M1-polarized macrophage and IL-1β. Mechanistic studies were further performed for evaluating whether CAB39/AMPK/Sirt1 pathway involves in OA progression.

### 3.1 CAB39 was down-regulated in the OA mouse model

**Figure 1.**
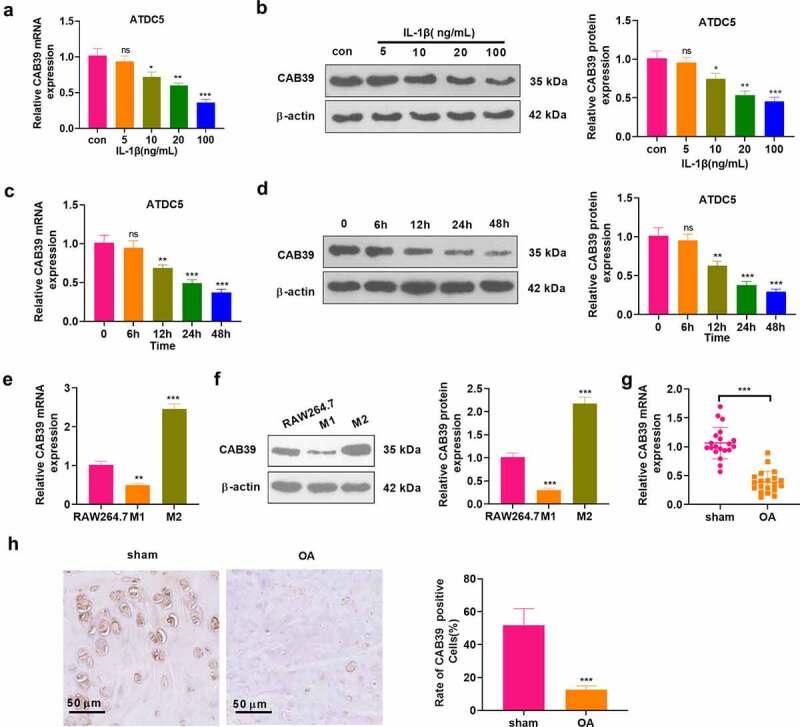
CAB39 was down-regulated in a mouse OA model.

To investigate the expression characteristics of CAB39 in cells and OA joint tissues, chondrocytes (ATDC5) were processed with varying concentrations of IL-1β (5, 10, 20, 100 ng/mL) for 24 hours. As indicated by qRT-PCR and WB, IL-1β hampered the CAB39 profile concentration-dependently (*P* < 0.05, Figure 1a-b). In parallel, chondrocytes (ATDC5) were treated with IL-1β (10 ng/mL) for 6, 12, 24, and 48 hours. qRT-PCR and WB were performed to test the CAB39 expression. As a result, IL-1β notably down-regulated CAB39 in a time-dependent manner (*P* < 0.05, Figure 1c-d). The M1 phenotype was induced by treating RAW264.7 cells with LPS (100 ng/mL) + IFNγ (20 ng/mL) for 24 hours. Meanwhile, IL-4 (20 ng/mL) + IL-13 (20 ng/mL) were adopted to treat RAW264.7 cells to induce the M2 phenotype. Expression of CAB39 in RAW264.7 cells and M1- and M2- polarized macrophages was testified by qRT-PCR and WB. Notably, CAB39 was distinctly down-regulated in M1-polarized macrophages and up-regulated in M2-polarized macrophages by contrast with that in normal RAW264.7 cells (*P* < 0.05, Figure 1e-f). Forty mice were taken and randomly divided into sham and OA groups (20 mice in each group). The mice in the experiment group were subjected to right knee DMM surgery to construct OA models and were executed four weeks after surgery to collect the right knee joint. qRT-PCR outcomes disclosed that the CAB39 expression was curbed in the OA group versus the sham group (*P* < 0.05, Figure 1g). IHC was applied to verify the CAB39 expression in mice’s joint tissues, revealing that there were significantly fewer CAB39-positive cells in the OA group versus the sham group (*P* < 0.05, Figure 1h). These outcomes disclosed that CAB39 was lowly expressed in both OA cellular and mouse models. We treated chondrocytes with 10 ng/mL IL-1β for 6, 12, 24, and 48 hours and observed a time-dependent decrease in CAB39 expression. Therefore, we used 10 ng/mL IL-1β to induce OA *in vitro* in the follow-up study.

Chondrocytes (ATDC5) were treated with varying concentrations of IL-1β (5–100 ng/mL) for 24 hours. A-B: The CAB39 expression was validated by qRT-PCR and WB. ATDC5 cells were treated with IL-1β (10 ng/mL) for 6, 12, 24, and 48 hours, respectively. C-D: qRT-PCR and WB gauged the CAB39 profile. M1 phenotype was induced in RAW264.7 cells by LPS (100 ng/mL) + IFNγ (20 ng/mL) treatment for 24 hours. Meanwhile, RAW264.7 cells were treated with IL-4 (20 ng/mL) + IL-13 (20 ng/mL) to induce the M2 phenotype. E-F: qRT-PCR and WB were conducted to check the CAB39 expression in RAW264.7 cells and M1- and M2-polarized macrophages. The mice were subjected to right knee DMM surgery to construct OA models and were executed four weeks after surgery to collect the right knee joint. G: The CAB39 profile in mouse joint tissues was ascertained by qRT-PCR. H: Immunohistochemical detection of CAB39-positive cell percentage in mouse joint tissues. Scale bar = 50 μm. ns *P* > 0.05, *P < 0.05, **P < 0.01, ***P < 0.001 (vs. con group), **P < 0.01, ***P < 0.001 (vs.RAW 264.7 group). N = 3 ***P < 0.001 (vs.sham group). N = 5

**3.2 Overexpressing CAB39 heightened the polarization of M1-phenotype macrophages to the M2 phenotype and attenuated IL-1β-mediated chondrocyte damage**

**Figure 2.**
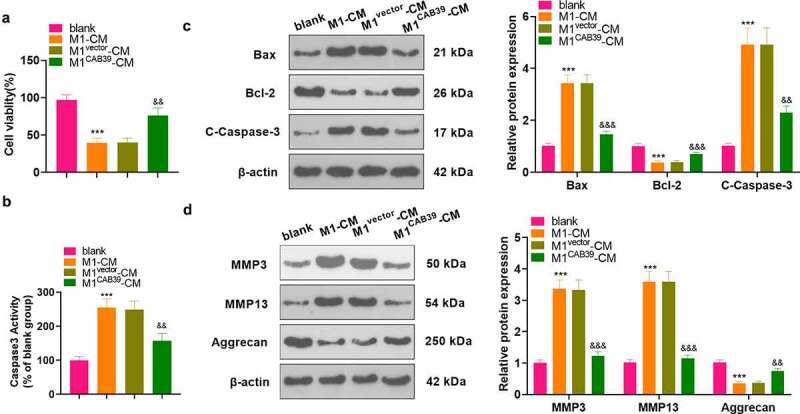
Overexpression of CAB39 facilitated M1 to M2 polarization of macrophages and attenuated IL-1β-mediated chondrocyte injury.

To figure out the impact of CAB39 on macrophage phenotype and chondrocytes, vectors and CAB39 overexpression plasmids were transfected into RAW264.7 cells. qRT-PCR was carried out to check the CAB39 profile, and the results displayed successful construction of CAB39 overexpression on macrophage and chondrocyte (*P* < 0.05, Figure 2a). The cells were treated with LPS (100 ng/mL) + IFNγ (20 ng/mL) and IL-4 (20 ng/mL) + IL-13 (20 ng/mL) to induce M1 and M2 phenotypes, respectively. The profiles of macrophage phenotypic markers (CD86, iNOS, CD206, and Arg1) were checked by WB. The data displayed that by contrast with the control group, the M1 group had increased CD86 and iNOS levels as well as reduced CD206 and Arg1 expression. Compared with the M1+ vector group, CAB39 overexpression suppressed CD86 and iNOS expression and heightened CD206 and Arg1 expression (*P* < 0.05, Figure 2b). In comparison to the control group, the expression of CD86 and iNOS was attenuated and the expression of CD206 and Arg1 was elevated in the M2 group. Additionally, the expression of CD86 and iNOS was impeded and that of CD206 and Arg1 was substantially heightened in the M2+ CAB39 group versus the M2+ vector group (*P* < 0.05, Figure 2c-d). Vectors and CAB39 overexpression plasmids were transfected into ATDC5 cells. qRT-PCR checked CAB39 expression in ATDC5 cells, revealing that the expression of CAB39 was boosted in the cells overexpressing CAB39 versus the vector group (*P* < 0.05, Figure 2e). ATDC5 cells were exposed to IL-1β (10 ng/mL). The CCK-8 data displayed that IL-1β abated cell viability versus the control group, and cell viability was substantially amplified after CAB39 overexpression versus the IL-1β+vector group (*P* < 0.05, figure 2f). Caspase3 activity detection results substantiated that IL-1β significantly intensified apoptosis versus the control group, and CAB39 overexpression dramatically hindered apoptosis versus the IL-1β+vector group (*P* < 0.05, Figure 2g). WB was employed for assessing the expression of apoptosis-related proteins (Bax, Bcl-2, and Caspase-3), MMP3, MMP13, and Aggrecan. The data disclosed that IL-1β augmented the expression of Bax, Caspase-3 cleavage, MMP3, and MMP13 and hampered the expression of Bcl-2 and Aggrecan versus the control group. Besides, by contrast with the IL-1β+vector group, CAB39 overexpression substantially down-regulated Bax, Caspase-3 cleavage, MMP3 and MMP13 and up-regulated Bcl-2 and Aggrecan (*P* < 0.05, Figure 2h-i). Thus, overexpression of CAB39 accelerated macrophages’ M1 phenotype toward the M2 phenotype and attenuated IL-1β-mediated chondrocyte injury.

Vectors and CAB39 overexpression plasmids were transferred into RAW264.7 cells. A: qRT-PCR tested the CAB39 profile. RAW264.7 cells were processed with LPS (100 ng/mL) + IFNγ (20 ng/mL) and IL-4 (20 ng/mL) + IL-13 (20 ng/m) to induce the M1 phenotype and M2 phenotype, respectively. B-D: WB was employed to evaluate the expression of CD86, iNOS, CD206, and Arg1. Vectors and CAB39 overexpression plasmids were transfected into ATDC5 cells. E: The CAB39 profile in ATDC5 cells was checked by qRT-PCR. ATDC5 cells were exposed to IL-1β (10 ng/mL). F: Cell viability was evaluated by CCK-8. G. Caspase3 activity detection kit checked cell apoptosis. H-I. The profiles of Bax, Bcl-2, Caspase-3, MMP3, MMP13, and Aggrecan were verified by WB. ***P < 0.001 (vs.vector group), *P < 0.05, **P < 0.01, ***P < 0.001 (vs.con group), ^&&^*P* < 0.01, ^&&&^*P* < 0.001 (vs.M1+ vector group), ^&&&^*P* < 0.001 (vs.M2+ vector group), ^&&^*P* < 0.01, ^&&&^*P* < 0.001 (vs.IL-1β+vector). N = 3

**3.3 Knocking down CAB39 abated the M2 phenotype of macrophages and intensified IL-1β-mediated chondrocyte injury**

**Figure 3.**
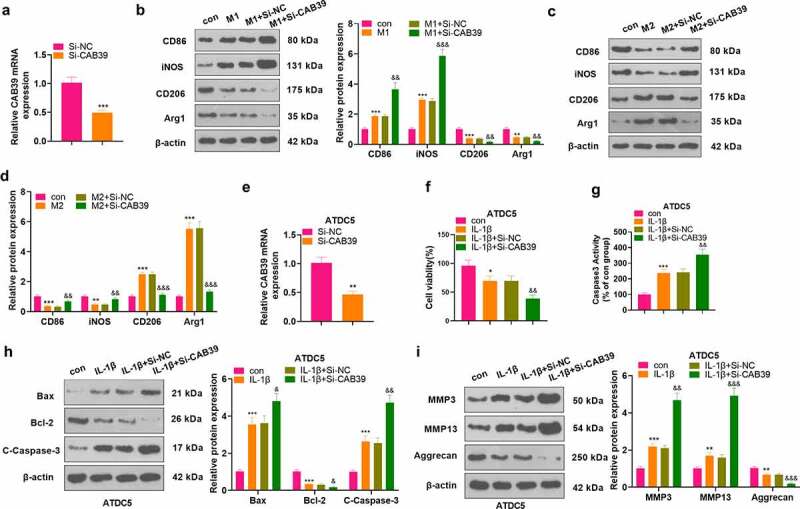
Knockdown of CAB39 curbed the M2 phenotype of macrophages and enhanced IL-1β-mediated chondrocyte injury.

Si-NC and Si-CAB39 were transfected into RAW264.7 cells. qRT-PCR testified that the CAB39 level was declined after knocking down CAB39 versus the Si-NC group (*P* < 0.05, Figure 3a). The cells were treated with LPS (100 ng/mL) + IFNγ (20 ng/mL) and IL-4 (20 ng/mL) + IL-13 (20 ng/mL) for 24 hours, respectively. The profiles of macrophage phenotypic markers (CD86, iNOS, CD206, and Arg1) were evaluated by WB. The data illustrated that knockdown of CAB39 markedly stimulated the expression of CD86 and iNOS and restrained the expression of CD206 and Arg1 as compared to the M1+ Si-NC group (*P* < 0.05, Figure 3b). In parallel, CD86 and iNOS were notably up-regulated and CD206 and Arg1 were remarkably down-regulated in the M2+ Si-CAB39 group versus the M2+ Si-NC group (*P* < 0.05, Figure 3c-d). Si-NC and Si-CAB39 were transfected into ATDC5 cells. As revealed by qRT-PCR outcomes, CAB39 was down-regulated after knockdown of CAB39 versus the Si-NC group (*P* < 0.05, Figure 3e). The above cells were exposed to IL-1β (10 ng/mL). CCK-8 data manifested that cell viability was visibly dampened following CAB39 knockdown versus the IL-1β+Si-NC group (*P* < 0.05, figure 3f). The Caspase3 activity detection result implied that knockdown of CAB39 resulted in enhanced apoptosis versus the IL-1β+Si-NC group (P < 0.05, Figure 3g). WB was applied to gauge the expression of Bax, Bcl-2, Caspase-3, MMP3, MMP13, and Aggrecan. As a result, compared to the IL-1β+Si-NC group, knockdown of CAB39 caused up-regulation of Bax, Caspase-3 cleavage, MMP3 and MMP13, accompanied by a diminished expression of Bcl-2 and Aggrecan (*P* < 0.05, Figure 3h-i). Hence, knocking down CAB39 restrained the M2 phenotype of macrophages and intensified IL-1β-mediated chondrocyte injury.

Si-NC and Si-CAB39 were transfected into RAW264.7 cells and A: qRT-PCR evaluated the CAB39 expression. The above cells were exposed to LPS (100 ng/mL) + IFNγ (20 ng/mL) and IL-4 (20 ng/mL) + IL-13 (20 ng/mL) to induce the M1 and M2 phenotype, respectively. B-D: The expression of CD86, iNOS, CD206, and Arg1 was measured by WB. Si-NC and Si-CAB39 were transfected into ATDC5 cells. E: qRT-PCR testified the CAB39 profile in ATDC5 cells. ATDC5 cells were treated with IL-1β (10 ng/mL). F: Cell viability was evaluated by CCK-8. G. Caspase3 activity detection kit checked cell apoptosis. H-I: WB was implemented for gauging the profiles of Bax, Bcl-2, Caspase-3, MMP3, MMP13, and Aggrecan. **P < 0.01, ***P < 0.001 (vs. Si-NC group), *P < 0.05, **P < 0.01, ***P < 0.001 (vs. con group), ^&&^*P* < 0.01, ^&&&^*P* < 0.001(vs. M1+ Si-NC group), ^&&^*P* < 0.01, ^&&&^*P* < 0.001 (vs. M2+ Si-NCgroup), ^&^*P* < 0.05, ^&&^*P* < 0.01, ^&&&^*P* < 0.001 (vs. IL-1β+Si-NC group). N = 3

### 3.4 Overexpression of CAB39 alleviated chondrocyte injury caused by macrophages

**Figure 4.**
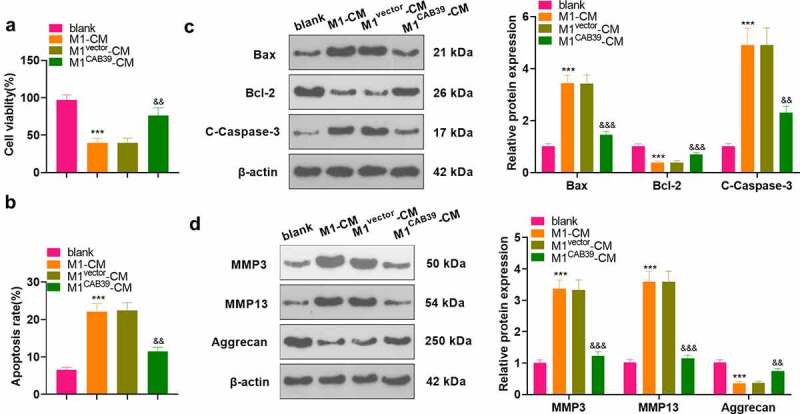
Overexpressing CAB39 alleviated chondrocyte injury induced by macrophages.

To probe the effect of CAB39 on macrophage-induced chondrocyte damage, vectors, and CAB39 overexpression plasmids were transfected into RAW264.7 cells, which were then treated with LPS (100 ng/mL) + IFNγ (20 ng/mL) for 24 hours. The CM of macrophages was harvested and co-cultured with ATDC5 cells for 24 hours. CCK-8 assayed ATDC5 cell viability and the data displayed that cell viability was notably reduced in the M1-CM group compared to the blank group and enhanced in the M1 ^CAB39^-CM group compared to the M1^vector^-CM group (P < 0.05, Figure 4a). Caspase3 activity test results demonstrated that the apoptosis rate was substantially higher in the M1-CM group than that of the blank group and lower in the M1^CAB39^-CM group compared to the M1^vector^-CM group (*P* < 0.05, Figure 4b). The expression of Bax, Bcl-2, Caspase-3, MMP3, MMP13, and Aggrecan was compared by WB, which exhibited that the profiles of Bax, Caspase-3 cleavage, MMP3, and MMP13 were facilitated and the expression of Bcl-2 and Aggrecan was curbed in the M1^CM^ group versus the blank group. The M1^CAB39-^^CM^ group had decreased Bax, Caspase-3 cleavage, MMP3 and MMP13 expression and increased Bcl-2 and Aggrecan contents versus the M1^vector^-CM group (*P* < 0.05, Figure 4c-d). Hence, overexpressing CAB39 attenuated macrophage-induced chondrocyte damage.

Vectors and CAB39 overexpression plasmids were transfected into RAW264.7 cells, which were intervened with LPS (100 ng/mL) + IFNγ (20 ng/mL) for 24 hours. The CM of macrophages was obtained and co-cultured with ATDC5 cells for 24 hours. A: The CCK-8 assay was applied for the ATDC5 cell viability test. B. Cell apoptosis was checked by Caspase3 activity detection kit. C-D: WB was implemented to verify the levels of Bax, Bcl-2, Caspase-3, MMP3, MMP13, and Aggrecan. *** *P* < 0.001 (vs.blank group), ^&&^*P* < 0.01, ^&&&^*P* < 0.001 (vs. M1 ^vector^ -CM group). N = 3

### 3.5 Overexpression of CAB39 promoted the AMPK/Sirt-1 axis

**Figure 5.**
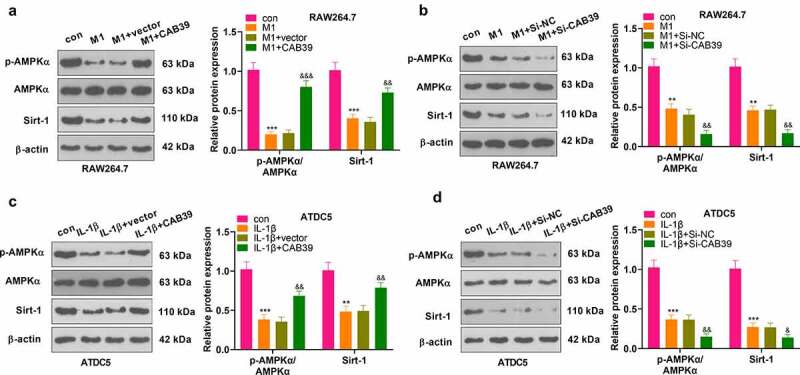
Overexpressing CAB39 motivated the AMPK/Sirt-1 pathway.

To make certain the mechanism of CAB39 in OA, vectors, CAB39 overexpression plasmids, Si-NC and Si-CAB39 were transfected into RAW264.7 and ATDC5. RAW264.7 cells were treated with LPS (100 ng/mL) + IFNγ (20 ng/mL), and ATDC5 cells were exposed to IL-1β (10 ng/mL) for 24 hours. The AMPK/Sirt-1 pathway expression was gauged by WB. The outcomes uncovered that the AMPK phosphorylation and Sirt-1 expression were reduced in RAW264.7 cells in the M1 group versus the control group, and they were significantly up-regulated following overexpression of CAB39 versus the M1+ vector group (*P* < 0.05, Figure 5a). On the contrary, the AMPK phosphorylation and Sirt-1 expression were hampered after the CAB39 knockdown versus the M1+ Si-NC group (*P* < 0.05, Figure 5b). In ATDC5 cells, IL-1β distinctly restrained AMPK phosphorylation and Sirt-1 expression. Contrarily, CAB39 overexpression enhanced AMPK phosphorylation and Sirt-1 expression versus the IL-1β+vector group (*P* < 0.05, Figure 5c). Moreover, compared to the IL-1β+Si-NC group, knockdown of CAB39 contributed to impaired AMPK phosphorylation and Sirt-1 expression (*P* < 0.05, Figure 5d). These results hinted that overexpressing CAB39 activated the AMPK and Sirt-1.

Vectors, CAB39 overexpression plasmids, Si-NC, and Si-CAB39 were transfected into RAW264.7 and ATDC5. RAW264.7 cells were processed with LPS (100 ng/mL) + IFNγ (20 ng/mL), while ATDC5 cells were exposed to IL-1β (10 ng/mL) for 24 hours. A-D: The AMPK/Sirt-1 pathway profile was ascertained by WB. **P < 0.01, ***P < 0.001 (vs.con group), ^&^*P* < 0.05, ^&&^*P* < 0.01, ^&&&^*P* < 0.001 (vs.M1+ vector group), ^&&^*P* < 0.01 (vs.M1+ Si-NC), ^&&^*P* < 0.01 (vs.IL-1β+vector), ^&^*P* < 0.05, ^&&^*P* < 0.01 (vs. L-1β+Si-NC group). N = 3

**3.6 Inhibition of the AMPK/Sirt-1 axis hindered the promotion of the M2 phenotype of macrophages and the protection of chondrocytes by CAB39 overexpression**

**Figure 6.**
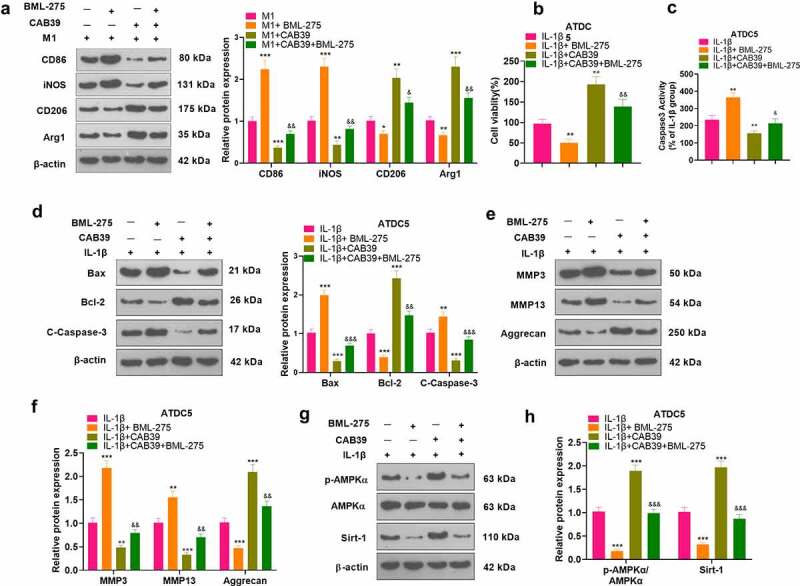
Inhibition of the AMPK/Sirt-1 pathway attenuated CAB39’s promoting effect on the M2 phenotype of macrophages and its protective effect on chondrocytes.

To further validate the mechanism of action of CAB39 in OA, vectors, and CAB39 overexpression plasmids were transferred into RAW264.7 cells. Next, the cells were exposed to LPS (100 ng/mL) + IFNγ (20 ng/mL) for 24 hours, followed by BML-275 (a potent and selective ATP-competitive AMPK inhibitor) (5 μM) intervention. WB was adopted to test the expression of macrophage phenotypic markers (CD86, iNOS, CD206, and Arg1). The outcomes disclosed that the addition of BML-275 in the M1 group resulted in up-regulation of CD86 and iNOS and down-regulation of CD206 and Arg1. In contrast, the addition of CAB39 in the M1 group led to a sharp decline in the expression of CD86 and iNOS and facilitation in the expression of CD206 and Arg1. Treatment with BML-275 distinctly augmented the expression of CD86 and iNOS and choked CD206 and Arg1 profiles versus the M1+ CAB39 group (*P* < 0.05, Figure 6a). Vectors and CAB39 overexpression plasmids were transfected into ATDC5 cells, which were processed with IL-1β (10 ng/mL) for 24 hours, followed by BML-275 (5 μM) treatment. As evidenced by CCK-8 data, the addition of BML-275 to the IL-1β group brought about a pronounced decrease in cell viability, and the addition of CAB39 to the IL-1β group led to an increase in cell viability. In parallel, cell viability was evidently weaker in the IL-1β+CAB39+ BML-275 group than that of the IL-1β+CAB39 group (*P* < 0.05, Figure 6b). Caspase3 activity examination data uncovered that the apoptotic rate was overtly higher in the IL-1β+ BML-275 group and lower in the IL-1β+ CAB39 group versus the IL-1β group. Meanwhile, the addition of BML-275 in the IL-1β+CAB39 group intensified apoptosis (*P* < 0.05, Figure 6c). The profiles of Bax, Bcl-2, Caspase-3, MMP3, MMP13, Aggrecan, and the AMPK/Sirt-1 pathway were testified by WB. The outcomes manifested that in comparison to the IL-1β group, Bax, Caspase-3 cleaved, MMP3, and MMP13 were notably up-regulated, while Bcl-2, Aggrecan, AMPK phosphorylation and Sirt-1 were down-regulated in the IL-1β+ BML-275 group. The opposite results were observed in the IL-1β+CAB39 group. Moreover, the IL-1β+CAB39+ BML-275 group had up-regulated Bax, Caspase-3 cleavage, MMP3, and MMP13 and down-regulated Bcl-2, Aggrecan, AMPK phosphorylation and Sirt-1 versus the IL-1β+CAB39 group (*P* < 0.05, Figure 6d-h). These outcomes confirmed that choking the AMPK/Sirt-1 pathway hindered CAB39 overexpression’s promoting effect on the M2 phenotype of macrophages and its protective effect on chondrocytes.

Vectors and CAB39 overexpression plasmids were transfected into RAW264.7 cells. RAW264.7 cells were treated with LPS (100 ng/mL) + IFNγ (20 ng/mL) for 24 hours, followed by BML-275 (5 μM) treatment. A: The profiles of CD86, iNOS, CD206, and Arg1 were testified by WB. Vectors and CAB39 overexpression plasmids were transfected into ATDC5 cells, which were then processed with IL-1β (10 ng/mL) for 24 hours, followed by BML-275 treatment. B: Cell viability was assessed by CCK-8. C: Apoptosis was gauged by Caspase3 activity detection kit. D-H: WB was carried out to monitor the expression of Bax, Bcl-2, Caspase-3, MMP3, MMP13, Aggrecan and the AMPK/Sirt-1 pathway. **P < 0.01, ***P < 0.001 (vs.M1group), **P < 0.01, ***P < 0.001 (vs.IL-1β group), ^&&^*P* < 0.01 (vs.M1+ CAB39 group), ^&&^*P* < 0.01, ^&&&^*P* < 0.001 (vs.IL-1β+CAB39 group). N = 3

### 3.7 Overexpressing CAB39 eased cartilage damage and inflammation in DMM mice

**Figure 7.**
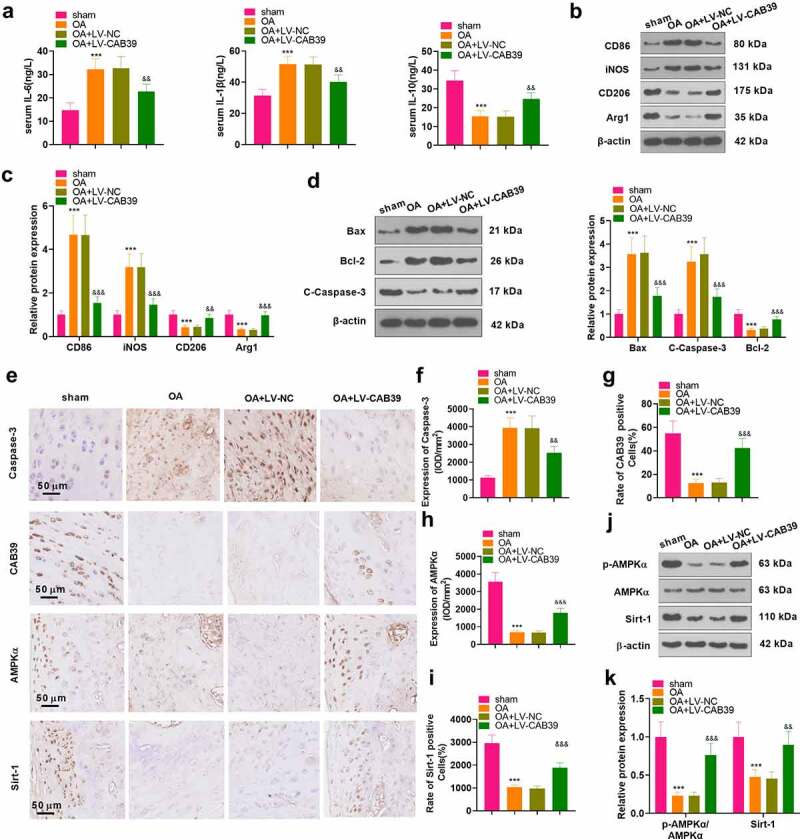
Overexpressing CAB39 eased cartilage damage and inflammation in DMM mice.

To inquire into the influence of CAB39 on OA *in vivo*, a mouse knee OA model was induced with DMM, and lentivirus encapsulated with vectors and CAB39 overexpression plasmids were administered to the right knee cavity of the mice. The mice’s blood and right knee joints were harvested. ELISA was implemented for measuring the expression of IL-6, IL-10 and IL-1β in mouse serum. The data exhibited that the levels of IL-6 and IL-1β were higher and those of IL-10 were lower in the OA group than those of the sham group. In parallel, the contents of IL-6 and IL-1β were significantly lower and those of IL-10 were higher in the OA+ LV-CAB39 group versus the OA+ LV-NC group (*P* < 0.05, Figure 7a). The expression of macrophage phenotypic markers (CD86, iNOS, CD206, and Arg1) and apoptosis-related proteins (Bax, Bcl-2, and Caspase-3) was checked by WB. The outcomes corroborated that CD86, iNOS, Bax and C-Caspase-3 were up-regulated, while CD206, Arg1 and Bcl-2 were down-regulated in the OA group versus the sham group. In contrast, overexpression of CAB39 depressed the expression of CD86, iNOS, Bax and C-Caspase-3 and fostered the expression of CD206, Arg1 and Bcl-2 (*P* < 0.05, Figure 7b-d). Expression of Caspase-3 and the CAB39/AMKP/Sirt-1 pathway in the knee synovium was monitored by IHC. As a result, the percentage of Caspase-3-positive cells was dramatically enlarged and the percentage of CAB39-, AMKP- and Sirt-1-positive cells was remarkably declined in the OA group versus the sham group. Nonetheless, overexpressing CAB39 obviously diminished the percentage of Caspase-3-positive cells and enlarged the percentage of CAB39-, AMPK- and Sirt-1-positive cells (*P* < 0.05, Figure 7e-i). Besides, we further validated the expression of the AMPP/Sirt-1 pathway with WB, and the outcomes were consistent with those of IHC. Namely, overexpressing CAB39 enhanced phosphorylation of AMPKα and expression of Sirt-1 (*P* < 0.05, Figure 7j-k). Thus, overexpressing CAB39 eased cartilage damage and inflammation in DMM mice.

DMM was performed to induce the mouse knee OA model, and lentiviruses coated with vectors and CAB39 overexpression plasmids were administered to the right knee cavity of mice. The mice’s blood and right knee joints were collected. A: ELISA was employed to test the expression of IL-6, IL-10 and IL-1β in mouse serum. B-D: WB estimated the expression of macrophage phenotypic markers (CD86, iNOS, CD206, and Arg1) and apoptosis-related proteins (Bax, Bcl-2, and Caspase-3). E-I: Immunohistochemical detection of Caspase-3 and the CAB39/AMPK/Sirt-1 pathway expression in medial interventricular cartilage of the knee joint. Scale bar = 50 μm. J-K: The AMPK/Sirt-1 pathway expression was assessed by WB. ***P < 0.001 (vs.sham group), ^&&^*P* < 0.01, ^&&&^*P* < 0.001 (vs.OA+LV-NC group). N = 5

## 4. Discussion

Here, we discovered that CAB39 was down-regulated in both OA mice and OA cell models. Overexpression of CAB39 activated the AMPK/Sirt-1 axis, boosted macrophages’ M1 polarization to M2 polarization, delayed the release of pro-inflammatory cytokines and attenuated IL-1β-mediated chondrocyte injury.

CAB39 contains two isoforms, CAB39α and CAB39β, and is an essential component of the LKB1-STRAD-CAB39 trimeric complex. The main role of CAB39 is to stabilize the binding of LKB1 and STRAD and to facilitate the translocation of LKB1 from the nucleus to the cytoplasm [34]. CAB39 overexpression triggers AMPK signaling activation [35]. Also, AMPK is a heterotrimeric complex comprising a catalytic α subunit and two regulatory β and γ subunits, and Thr172 phosphorylation in the α subunit of AMPK is the main mechanism of AMPK activation [36]. According to reports, the combination of LKB1 and STRAD-CAB39 activates AMPK through phosphorylation of Thr172 [37]. AMPK plays a vital role in modulating immune cell metabolism and inflammation. The NOD-like receptor pyrin domain 3 (NLPR3) inflammasome mediates IL-1β production with the requirement to activate the metabolite ROS, while activation of AMPK diminishes mitochondrial ROS production to curb NLPR3 inflammasome activation [38]. AMPK exhibits potent anti-inflammatory activities by indirectly choking NF-κB through activation of Sirt-1 and forkhead box O (FOXO) [39]. A recent study has revealed that d-Mannose heightens chondrocyte proliferation and reduces apoptosis via the AMPK pathway activation [40]. Sirt-1 is a major downstream effector of AMPK that deacetylates the NF-κB p65 subunit to induce inactivation of the NF-κB pathway [41]. Sirt-1 plays a key role in OA, and low Sirt-1 expression fosters chondrocyte apoptosis by regulating the mitochondrial apoptotic pathway [42]. Shi et al. have demonstrated that lncRNA MCM3AP-AS1 up-regulates Sirt-1 expression and attenuates OA chondrocyte damage by targeting miR-138-5p [43]. Besides, peroxisome proliferator-activated receptor-γ (PPARγ) agonist restrains the inflammatory response in OA chondrocytes through the activation of the AMPK/Sirt-1 pathway [44]. In this study, we substantiated that overexpression of CAB39 enhanced AMPKα phosphorylation and Sirt-1 expression and attenuated chondrocyte apoptosis induced by IL-1β. Meanwhile, AMPK inhibitors reversed the protective effect of CAB39 overexpression on chondrocytes. *In vivo*, overexpressing CAB39 declined the number of CD86-labeled M1-polarized macrophages and increased the number of CD206-labeled M2-polarized macrophages in synovial tissues, ameliorating cartilage damage and inflammation in OA model mice.

Previous studies have shown that chondrocyte apoptosis and extracellular matrix (ECM) degradation are key evidence of OA progression. Caspases are important molecules that regulate apoptosis, with caspase-3 being the most critical one. Caspase-3 has been reported to induce classical apoptosis by contributing to DNA breakage and cell death in a variety of tissues and cells, including cartilage [45]. Intracellular apoptosis is also regulated by mitochondrial function. The B-cell lymphoma-2 (Bcl-2) family modulates the mitochondrial apoptotic pathway by regulating the permeability of the mitochondrial membrane. The balance between the anti-apoptotic protein Bcl-2 and the pro-apoptotic protein Bcl-2-associated X protein (Bax) controls apoptosis [46]. It has been reported that sodium nitroprusside induces apoptosis in human chondrocytes and that Caspase-3 expression is up-regulated and Bcl-2 expression is down-regulated in sodium nitroprusside-treated chondrocytes [47]. Thus, Caspase-3, Bcl-2 and Bax can be used as biomarkers of chondrocyte apoptosis in OA [46]. In addition, increased degradation and decreased synthesis of ECM is one of the main causes of articular cartilage destruction in OA [48]. MMP3 and MMP13 have been implicated in fostering ECM degradation [49–51]. Hu et al. established that Aggrecan can enhance cartilage tissue formation [52] and that MMP drives Aggrecan degradation and exacerbates OA by cleaving Aggrecan [53]. In the present study, we observed that IL-1β treatment boosted the expression of Caspase-3, Bax, MMP3 and MMP13 in chondrocytes, while overexpression of CAB39 markedly depressed the expression of the above proteins and up-regulated Bcl-2 and Aggrecan. In addition, the use of BML-275 further up-regulated IL-1β-induced Caspase-3, Bax, MMP3, and MMP13, and restrained the levels of Bcl-2 and Aggrecan. The above results demonstrate that CAB39 mitigates IL-1β-mediated chondrocyte injury by suppressing chondrocyte apoptosis and extracellular matrix degradation through activation of the AMPK/Sirt-1 pathway.

Macrophages express a variety of cell surface receptors that are triggered by specific stimuli, which further polarize macrophages into M1 or M2 types. Macrophages exhibit the M1 phenotype when activated by LPS and IFN-γ or TNF-α, showing high expression of co-stimulatory CD80/86 molecules (e.g. CD86), reactive oxygen species and nitrogen species (e.g. iNOS) [54,55]. M2 macrophage polarization is induced by a variety of stimuli, such as IL-4 alone or in combination with IL-13, toll-like receptors or IL-10. M2 macrophages secrete anti-inflammatory cytokines upon activation and highly express several surface receptors, such as CD206 and CD163 [56]. Our data indicated that CD86 and iNOS were up-regulated when the M1 phenotype was induced using LPS+IFN-γ. In parallel, the expression of CD206 and Arg1 was facilitated when the M2 phenotype was induced using IL-4+ IL-13. In addition, overexpression of CAB39 enhanced the expression of M2 phenotype markers and suppressed the expression of M1 phenotype markers. Chondrocytes were markedly depressed in viability and enhanced in apoptosis after co-culture with CM of M1-polarized macrophages. Synovial macrophage activation plays an important role in the progression of OA. Macrophages are accumulated and polarized (M1 or M2) in the synovial membrane and joint cavity during OA. It has been testified that M1-polarized macrophages but not M2-polarized macrophages are clustered in human and mouse OA synovial tissues. M1 polarization of synovial macrophages exacerbates the progression of OA in the DMM model mice, while M2 polarization retards OA progression [57]. Here, we demonstrated that overexpression of CAB39 heightened M1 to M2 polarization of macrophages and attenuated macrophage-induced chondrocyte damage.

## Conclusion

Macrophage inflammation and the release of pro-inflammatory cytokines are crucial mechanisms of OA evolvement. Here, we found for the first time that CAB39 enhanced macrophages’ M2 polarization and ameliorated OA-like chondrocyte injury by motivating the AMPK/Sirt-1 pathway in macrophages and chondrocytes. Therefore, CAB39 may be a potential molecular target for OA treatment [58].

## Data Availability

The data sets used and analyzed during the current study are available from the corresponding author on reasonable request.
